# Congenital Heart Defect and Pulmonary Hypertension in Children With Down Syndrome: Clinical Profile Over Two Decades

**DOI:** 10.7759/cureus.13212

**Published:** 2021-02-07

**Authors:** Abdullah N Alhuzaimi, Najoud M Alotaibi, Ghadah I Alsuhaibani, Reem K Alanazi, Mohamad-Hani Temsah

**Affiliations:** 1 Pediatric Cardiology, Department of Cardiac Science, College of Medicine, King Saud University Medical City, King Saud University, Riyadh, SAU; 2 Pediatric Intensive Care Unit, Department of Pediatrics, College of Medicine, King Saud University Medical City, King Saud University, Riyadh, SAU

**Keywords:** down syndrome, trisomy 21, congenital heart defect, pulmonary hypertension, prevalence

## Abstract

Objectives: To describe the frequency and spectrum of congenital heart defects (CHD) and pulmonary hypertension among pediatric patients with Down syndrome (DS) in Saudi Arabia.

Methods: A cross-sectional, retrospective study of the cardiac anomalies among pediatric patients (0-18 years) with DS had been seen and evaluated in one center from August 2001 to October 2020. The demographic data, the reason for referral, echocardiography data including presence and type of CHD, systolic function, atrioventricular regurgitation, and pulmonary hypertension (PHTN) were analyzed.

Results: Among the 468 pediatric patients with DS, 275 (58.8%) had one or more congenital heart defects (CHD). The most common types of CHD among DS pediatric patients were ventricular septal defect (29.45%), atrial septal defect (ASD) secundum (26.9%) and atrioventricular septal defect (AVSD) (22.9%), and moderate to large patent ductus arteriosus (PDA) (9.1%). Pulmonary hypertension analyzed in children older than two months of age and was present in 21.5% of patients with CHD and 2.2% of patients with no CHD. Multivariate logistic regression showed the presence of AVSD, large PDA, and ASD secundum which all independent predictors of pulmonary hypertension.

Conclusion: Almost 60% of DS patients have CHD with pulmonary hypertension which affect almost one-fifth of patients with CHD. AVSD, hemodynamically significant PDA, and ASD secundum were the most common lesions associated with pulmonary hypertension.

## Introduction

Down syndrome (DS) is the most common chromosomal abnormality worldwide. It occurs in one of every 733 live births in the US [1]. It occurs due to a non-disjunction mechanism in most cases leading to an extra copy of chromosome 21 [2]. Children with DS are usually diagnosed during the neonatal period based on their distinctive dysmorphic features. The syndrome is characterized by ﻿cognitive impairment, facial dysmorphism, generalized hypotonia, and other congenital abnormalities, including cardiac and gastrointestinal anomalies [3,4].

Almost one-half of patients with Down syndrome have congenital heart defects (CHD) [3,4]. There is limited data on the prevalence and clinical spectrum of CHD and pulmonary hypertension among Saudi Down syndrome patients [5,6]. CHD is the second cause of mortality among patients with Down syndrome; therefore, early diagnosis and management are warranted to prevent the poor outcome [7].

We aimed in this study is to describe the profile of CHD and pulmonary hypertension among children with Down syndrome over two decades in a single center in Saudi Arabia.

## Materials and methods

We conducted a retrospective cross-sectional descriptive study of the cardiac defects among pediatric DS patients (0-18 years) who were seen in King Khalid University Hospital (KKUH), Riyadh, from August 2001 till December 2019. The hospital is an academic multi-disciplinary facility that provides primary, secondary, and tertiary care with busy obstetrics services. The hospital is covered with in house pediatric cardiology diagnostic services. The study was approved by the institutional review board (Approval # E-20-4907). The patients were identified and data were collected through the electronic database of pediatric cardiology and the echocardiography database, which contains the clinical and echocardiographic data. A total of 468 patients who met the inclusion criteria were included.

Medical records and echocardiography reports were reviewed for the following data: reasons of referral, date of echocardiography, age at the time of referral, gender, weight, height, presence or absence of congenital heart defect CHD, type and details of CHD, presence or absence of pulmonary hypertension (PHTN), blood pressure in pulmonary hypertension patients, presence and degree of valvular abnormalities, history of intervention (cardiac surgeries or trans-catheter interventions).

Left ventricular (LV) systolic function parameters were obtained from M-Mode echocardiography; the ejection fraction (EF) and shortening fraction (SF) with normal value for EF is > 60% and SF > 30% according to international standards. In patients who had a surgical repair, we looked to their last echocardiography for any residual lesions. Pulmonary hypertension is diagnosed if the estimated systolic pulmonary artery pressure exceeds 30 mmHg [8]. The patients were diagnosed with pulmonary hypertension based on their echocardiographic assessment by determining peak tricuspid regurgitation or shunt across ventricular septal defect (VSD) or patent ductus arteriosus (PDA) if present. The pulmonary artery systolic pressure was assumed to be equal to the tricuspid regurgitation (TR) gradient plus estimated mean right atrial pressure of 5 mmHg. In the presence of VSD or PDA, the pulmonary artery systolic pressure was estimated based on the following formula: pulmonary artery systolic pressure = aortic systolic pressure - VSD or PDA peak left to right gradient. If there was a systolic right to left shunt across VSD or PDA, this was considered diagnostic for pulmonary hypertension. The outcome for pulmonary hypertension was analyzed for patients older than two months to avoid over-diagnosing patients during the transient normal drop of pulmonary artery pressure in the early postnatal stage. The patients with small PDA or small atrial septal defect (ASD) II were not accounted for if the PDA closed spontaneously over the first six months. 

The data were analyzed for descriptive analytics such as frequency, mean, and standard deviation, and for categorical data, a chi-square test was applied, while a student-test was used for numerical data.

For data analysis, statistics conducted included means, medians, and proportions were used to describe patients' characteristics. Binary group comparisons were made using T-test, Chi-Square, and Mann-Whitney tests as appropriate. Statistical analysis was done using Statistical Package for the Social Sciences (SPSS) version 21 for Windows 8.1 (IBM Corp., Armonk, NY, USA).

## Results

**Figure 1:**
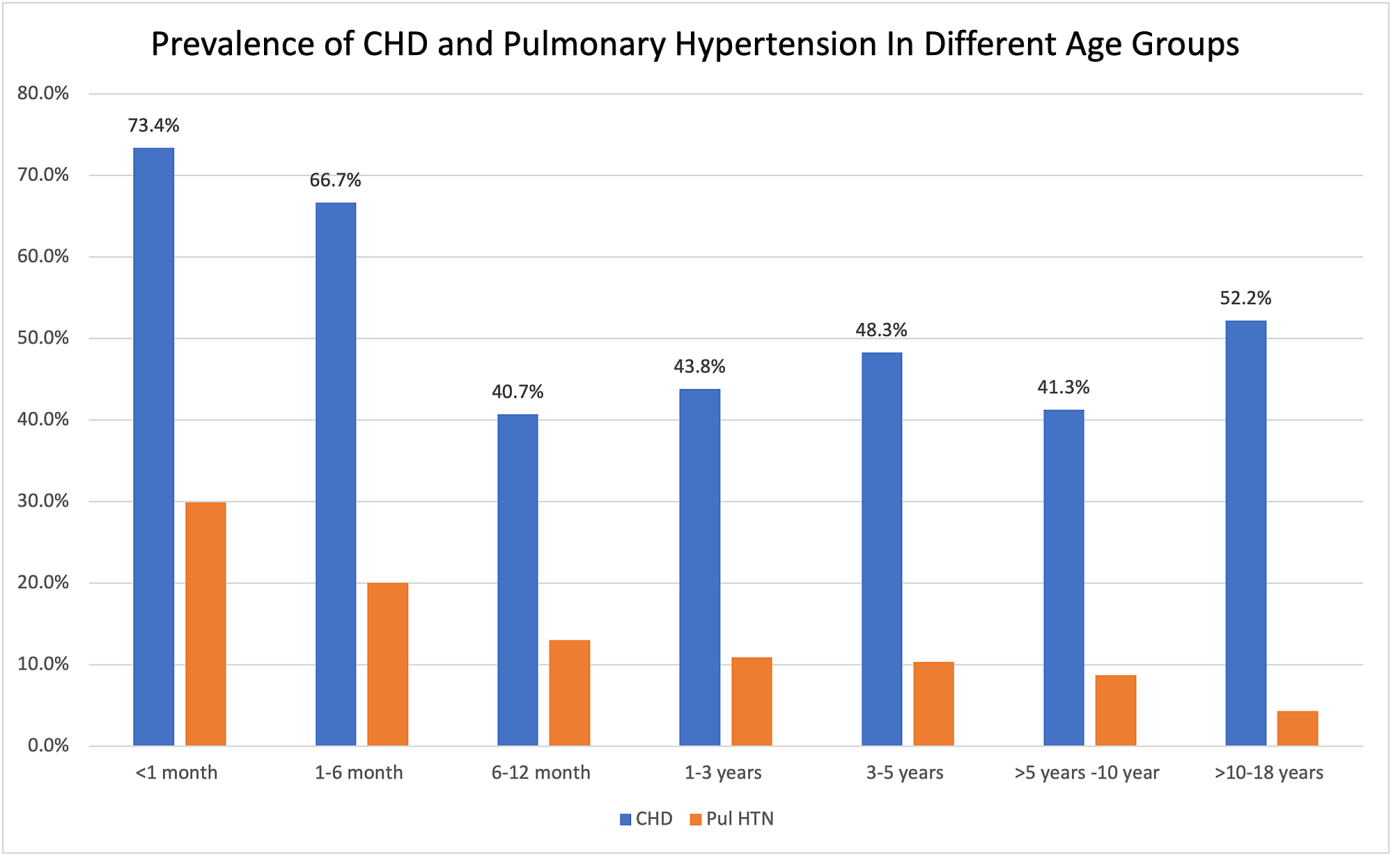
Prevalence of Congenital Heart Defects (CHD) and Pulmonary Hypertension In Different Age Groups

Among the 468 pediatric patients with DS, 275 had one or more CHD. The median age at the first assessment was five months (range 0-18 years). The median weight was 4.9 kg (range 1.1 kg-119 kg), and the median height was 58.4 cm (range 35 - 154 cm). More than half of the study population were evaluated at or less than the age of six months. The prevalence of CHD and pulmonary hypertension is highest among younger patients and decreases in older age groups (Figure 1).

**Figure 2:**
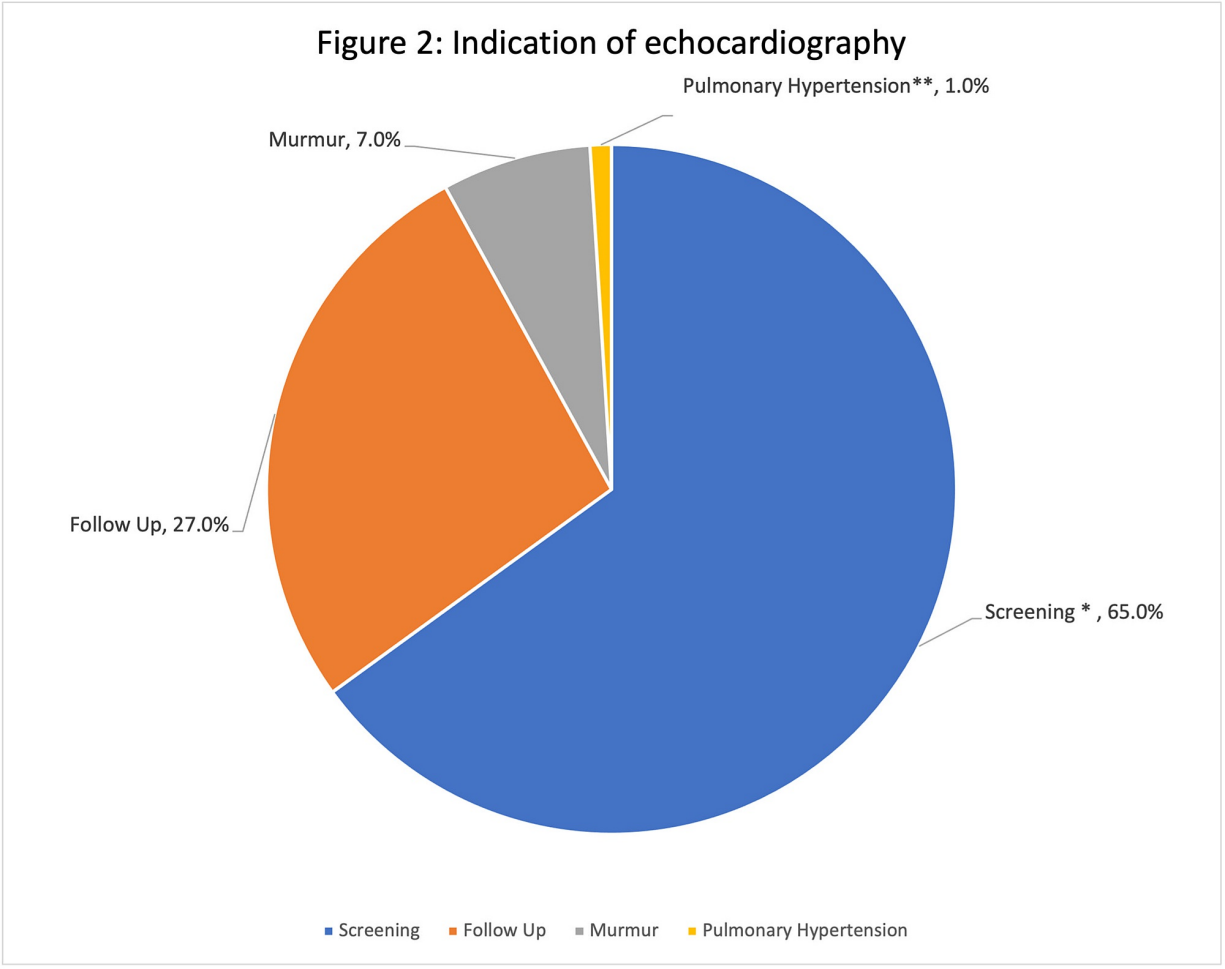
Indications of echocardiography _* Screening protocol for all newborn with clinical features of Down syndrome _ _** Either known case of pulmonary hypertension or to rule out pulmonary hypertension  _

All patients with DS features who were born in our hospital underwent initial echocardiography screening. The indications of echocardiography for the whole patient cohort are shown in Figure 2. The most common indication for echocardiography was for initial screening or follow up of already diagnosed cardiac disease. Only 1% of patients had echocardiography to follow a diagnosed pulmonary hypertension or to rule it out. 

Patients with CHD were significantly younger, with a lower weight (P-value < 0.05) and height (P-value < 0.05) compared to patients with no CHD (Table 1). The frequency for different CHD occurrences among DS patients is depicted in Table 2. The prevalence of different CHD per 100 DS patients ranged from <1% to 22%.

**Table 1:** Down Syndrome Patient Characteristics CHD: congenital heart defects

	CHD (n=275) Mean ±SD	No CHD (n=193) Mean ±SD	Total (n=468) Mean ±SD	P-value
Age at first assessment (years)	1.59±3.15	2.71 ±3.53	2.06±3.36	0.003
Weight (kg)	7.75±10.29	13.10±16.29	9.95±13.3	<0.001
Height (cm)	64.09±24.97	76.69±28.01	69.29±26.96	0.01
Gender (male)	152 (55.3%)	105 (54.4%)	257 (54.9%)	0.853
Age at last visit (years)	3.54±4.01	4.83±4.46	3.91±4.17	0.143
Pulmonary Hypertension	84 (30.5%)	6 (3.1%)	90 (19.2%)	<0.001
Fractional shortening %	38.75±6.41	38.8±7.18	38.78±6.87	0.134

**Table 2:** Frequency and Prevalence of Different CHD Among Down Syndrome Patients PDA: patent ductus arteriosus, CHD: congenital heart defects, VSD: ventricular septal defect, AVSD: atrioventricular septal defect, TOF: tetralogy of Fallot, DORV: double outlet right ventricle, ASD: atrial septal defect, PA: pulmonary arteries

CHD Breakdown	Frequency of cases	Prevalence per 100 Downs syndrome patients
PDA (hemodynamically significant)	57	12
VSDs (all expect AVSD, TOF, and DORV)	81	17
muscular	31	7
Inlet	5	1
Perimembranous	45	10
ASD II total		
ASD Secundum	102	22
ASD II with AVSD	10	2
AVSD	63	13
Complete AVSD (Primum ASD, VSD, mitral valve cleft)	58	12
Partial AVSD (Primum ASD +Cleft MV)_	5	1
Other CHD :		
TOF	7	1
DORV	2	0
Coarctation of Aorta	7	1
Mitral valves prolpase	3	1
Left superior vena cava	4	1
Dysplastic Tricuspid valve	1	0
Subaortic membrane with PDA	1	0
Peripheral PA stenosis	6	1
Pulmonary stenosis	2	0
Aortic stenosis	1	0
Straddling tricuspid valve with VSD	1	0
Bicuspid aortic valve	9	2
Right arch with aberrant left subclavian	2	0

The distribution of CHD for all patients with CHD is displayed in Table 3. The most common CHD among DS patients are shown in Table 3. The most common CHD were ventricular septal defect (29.45%), ASD secundum (26.9%), and atrioventricular septal defect (AVSD) (22.9%). The CHD type classification was placed under the most significant lesions, and if the patient has AVSD and additional ASD II or PDA, then these were classified under AVSD in Table 3. 

**Table 3:** Distribution of CHD (N=275) * (arranged from most common to least common) PDA: patent ductus arteriosus, CHD: congenital heart defects, CoA: coarctation of the aorta, VSD: ventricular septal defect, AVSD: atrioventricular septal defect, TOF: tetralogy of Fallot, DORV: double outlet right ventricle, BAV: bicuspid aortic valve, ASD: atrial septal defect, LPA: left pulmonary arteries, TV: tricuspid valve

Distribution of CHD (N=275) *	Number	Percentage ( %)
VSD	81	29.45
muscular	31	11.27
Inlet	5	1.82
Perimembranous	45	16.36
ASD II Isolated	74	26.91
AVSD	63	22.91
AVSD complete (Primium ASD, VSD,cleft)	49	17.82
AVSD Partial (Primium ASD +Cleft MV)_	5	1.82
AVSD with TOF or DORV (one with vascular ring)	4	1.45
AVSD with PS	1	0.36
AVSD with BAV	1	0.36
AVSD with CoA	3	1.09
PDA	25	9.09
PDA with ASD II	15	5.45
TOF or DORV	5	1.82
Coarctation of Aorta	4	1.45
Isolated Peripheral pulmonary artery stenosis	2	0.73
Bicuspid aortic valve	2	0.73
Vascular ring	1	0.36
aortic stenosis	1	0.36
Dysplastic TV and LPA stenosis	1	0.36
Subaortic membrane	1	0.36
		Total = 100

Pulmonary hypertension

The presence of pulmonary hypertension was analyzed in infants older than two months. Pulmonary hypertension was present among 31/268 (11.6%) patients, 28/130 (21.5%) patients had CHD, and only 3/138 (2.2%) patients did not have CHD. The odds ratio of pulmonary hypertension in CHD patients was 12.3 times (CI 3.65-41.76, P-value < 0.05) higher than patients without CHD. The prevalence of pulmonary hypertension was noted to be highest among patients with AVSD (34.4%), followed by hemodynamically significant PDA (36.4%), followed by ASD secundum (22.9%), then VSD (12.2%).

Multivariate binary logistic regression was conducted (Table 4) to assess the risk of pulmonary hypertension among patients older than two months. Five independent variables were fitted to test the relationship between the likelihood of pulmonary hypertension in the presence of different CHD categories. Almost 23.7% of variance depend on this model (Nagelkerke R2 = 0.237, Hosmer and Lemeshow Test P = 0.81). The presence of AVSD and hemodynamically significant PDA were significantly associated with pulmonary hypertension with high odds ratio (OR 12.2, P < 0.05) (OR 9.3, P < 0.05) consecutively. Patients with ASD secundum were 5.3 times (OR 5.3, P < 0.05) more likely to have pulmonary hypertension. Patient age and the presence of VSD were not significant predictors of pulmonary hypertension.

**Table 4:** Multivariate Binary Logistic Regression Analysis of Pulmonary Hypertension* in Down Syndrome Patients Older Than Two Months N=268 Dependent variable = pulmonary hypertension PDA: patent ductus arteriosus, VSD: ventricular septal defect, AVSD: atrioventricular septal defect, ASD: atrial septal defect

Table 4: Multivariate Binary Logistic Regression Analysis of Pulmonary Hypertension* in Down Syndrome Patient older than Two Months N=268				
	Exp(B)	95% CI for EXP(B)		P value
		Lower	Upper	
Patient Age	0.93	0.818	1.057	0.267
ASD II	5.342	2.038	14.001	0.001
AVSD	12.203	4.224	35.254	< 0.001
VSD	2.256	0.709	7.178	0.168
PDA	9.317	2.046	42.427	0.004
Constant	0.047			0

Atrioventricular valves abnormalities

Mitral regurgitation (MR) was present in 16.7% of DS patients with CHD (46/275) and 5.7% (11 patients) with no CHD. Moderate to severe MR was associated with the presence of CHD, especially AVSD (Table 5). Tricuspid regurgitation (TR) was present in 41.1% of patients with CHD (112/275), with 28 patients having moderate to severe TR. On the other hand, both MR and TR were much less common in the No-CHD group, with a prevalence of MR and TR of 5.7% (11/193) and 10.4% (20/193) consecutively. Semilunar valve abnormalities are depicted in (Tables 2) and (Table 3).

**Table 5:** Additional Details of Other Cardiac Abnormalities MR: mitral regurgitation, TR: tricuspid regurgitation, VSD: ventricular septal defect, AVSD: atrioventricular septal defect, CHD: congenital heart defects, TV: tricuspid valve

Other Cardiac abnormalities	Frequency	Details
Pericardial effusion	13	6 with CHDs, 3 moderate , 10 mild /small
MR (35 of them has AVSD)	57	46 has CHDs
mild	38	28 has CHD, 22 has AVSD
moderate	18	17 has CHD, 13 has AVSD
severe	1	1 has CHD, not AVSD
TR	133	113 has CHD
mild	105	23 has AVSD, 87 has CHD
moderate	26	13 has AVSD, 24 has CHD
severe	2	2 has CHD, one AVSD, and another TV dysplasia
LV dysfunction	4	2 has CHD post-op, 1 has small VSD

## Discussion

Our study is the largest study addressing CHD among Down syndrome patients in Saudi Arabia. The incidence of DS in Saudi Arabia was estimated to be 1.8/1000 live births [9]. Almost 60% of our Down syndrome population were affected by one or more congenital heart defects. The incidence of CHD was higher than similar international studies. Freeman et al. showed an incidence of 45% of CHD among Down syndrome patients in a population-based study done in Atlanta, USA [10]. Another study done in Libya showed a similar proportion of patients with Down syndrome who had CHD [11]. The sensitivity to detect CHD diagnosis has increased over the years, with the detection rate increased from 20% in the early 1970s to more than 50% in the late 1980s [12].

There is a regional variation in the incidence and types of CHD from different regions in Saudi Arabia [6]. A similar but smaller study was done in the central region of Riyadh and showed almost 45% of the patient population have a CHD [13]. Variable inclusion of minor cardiac lesions might add to the variability in incidence of CHD in different studies. We included small PDA or ASD secundum only if they persisted over multiple visits. This may explain our lower prevalence of PDA at 12%. The most common CHD in our series were ventricular septal defect followed by ASD secundum, then AVSD. There is significant variation in the commonest cardiac lesions, with older studies showing AVSD as the most common cardiac lesion reaching up to 30-54% in various studies in Europe and the USA [14-18]. Although AVSD is not the most common cardiac lesion in our series, it is the most common significant lesion requiring intervention. Atrioventricular valve regurgitation was highly prevalent among DS patients. Tricuspid regurgitation was observed in 55.7% of the fetuses with trisomies 21 [19]. And almost 40% of patients with CHD were found to have tricuspid regurgitation.

Down syndrome is strongly linked with pulmonary hypertension. The presence of upper airway obstruction and congenital heart disease are both significant factors for the development of pulmonary hypertension [20]. The prevalence of pulmonary hypertension is 2.2% among our down syndrome population with no CHD and 21.5% among patients with CHD in our population. A similar study showed up to 28% of all patients with Down syndrome have pulmonary hypertension; 70% have a transient form of pulmonary hypertension, and the majority have persistent pulmonary hypertension of the newborn [21]. 

Persistent exposure to increased pulmonary blood flow due to left to right shunt lesions (e.g., ventricular septal defect) may result in vascular remodeling and dysfunction leading to increased pulmonary vascular resistance and pulmonary hypertension [22]. Advanced degree of pulmonary hypertension secondary to CHD can lead to a right to left shunt and development of Eisenmenger’s syndrome. Almost half of adult patients with Eisenmenger’s syndrome have Down syndrome [23]. Therefore, early and timely diagnosis and surgical repair are needed to avoid pulmonary hypertension. The risk of pulmonary hypertension varies with the type and size of cardiac lesions. Dutch national registry of congenital heart disease showed a prevalence of pulmonary hypertension among adult patients with CHD of 4.2%. The risk of pulmonary hypertension was 8% among patients with simple ASD, 11% among patients with VSD, and up to 41% among patients with AVSD with either closed or non-closed defects [24]. Our data showed pulmonary hypertension was highest among patients with AVSD and PDA.

It is important to mention few limitations to this study; the reported frequency of CHD was based on retrospective data and reflects the population seen in our echocardiography laboratory and might have missed patients who never had echocardiography in our hospital. Also, we could not include the surgical or interventional outcome as cases that needed intervention were referred to other centers for intervention.

## Conclusions

Almost 60% of Down syndrome patients have one or more CHD types, excluding minor small ASD secundum and small PDA. The most common CHDs are ventricular septal defect, ASD secundum, and AVSD. Pulmonary hypertension is highly associated with AVSD, hemodynamically significant PDA, and ASD secundum. However, in the era of early diagnosis and timely repair of CHD, the risk of pulmonary hypertension is low.
